# Identification of *Candida* spp. isolated from oral mucosa in patients with leukemias and lymphomas in Iran

**Published:** 2019-04

**Authors:** Sara Hamzehee, Davood Kalantar-Neyestanaki, Mohammad Ali Mohammadi, Saeed Nasibi, Seyed Amin Ayatollahi Mousavi

**Affiliations:** 1 Student Research Committee, Department of Parasitology and Mycology, School of Medicine, Kerman University of Medical Sciences, Kerman, Iran; 2 Department of Microbiology and Virology, School of Medicine, Kerman University of Medical Sciences, Kerman, Iran; 3 Research Center for Hydatid Disease in Iran, Kerman University of Medical Sciences, Kerman, Iran; 4 Department of Medical Mycology and Parasitology, School of Medicine, Kerman University of Medical Sciences, Kerman, Iran

**Keywords:** *Candida* spp., Oral candidiasis, Hematologic malignancies, Lymphomas

## Abstract

**Background and Objectives:**

Oral candidiasis is a serious problem for immunocompromised patients, especially patients with hematological malignancies. After becoming a systemic candidiasis it is difficult to diagnose, control and treat in individuals with hematological malignancies. The aim of this study was to diagnose candidiasis in the oral mucosa of patients with leukemias and lymphomas in a timely manner in order to prevent their progression to systemic candidiasis.

**Materials and Methods:**

In this cross sectional study, 50 clinical samples were collected from the mouth of patients with hematological malignancies undergoing chemotherapy from the oncology units of teaching hospitals in Kerman, Iran. Patients were from Kerman, Sistan-Baluchestan and Hormozgan in south-eastern Iran. Sampling was restricted to patients with diagnosed acute lymphoid leukemia (ALL); acute myeloid leukemia (AML); chronic lymphoid leukemia (CLL); chronic myeloid leukemia (CML); Hodgkin's lymphoma (HL) and non-Hodgkin's lymphoma (NHL). Presumptive species identification of fungi was performed using conventional methods like colony characteristics on CHROMagar *Candida* medium, germ tube production, and assessing the morphology fungi on corn meal agar. Confirmation of presumptive candida isolates was performed using PCR-RFLP.

**Results:**

From a total of 50, 14 patients (28%) had positive oral candidiasis. *Candida albicans* (57.14%) was the most common species followed by *Candida glabrata* (14.28%), *Candida parapsilosis* (14.28%), *Candida krusei* (7.14%) and *Candida kefyr* (7.14%). *Candida albicans* had the highest rate of oral infection in ALL (35.71%) and then NHL (28.57%) patients.

**Conclusion:**

The results indicate that oral candidiasis is a prevalent fungal infection in the patients with hematologic malignancies with *C. albicans* being the main etiological agent. However, other species of *Candida* cause similar infections in these patients.

## INTRODUCTION

*Candida* species are opportunistic fungi. The most cases of candidiasis are caused by *C. albicans* followed by *C. glabrata, C. parapsilosis, C. tropicalis, C. krusei* and *C. kefyr* (1). Oral candidiasis is one of the most important forms of mucosal candidiasis. A weakened mucosal barrier permits translocation of fungi present in the mouth, oropharynx, gastro-intestinal tract or skin to enter the body and, when this is combined with immunosuppression, severe infections are common (2). The incidence of opportunistic fungal infections such as oral candidiasis has increased in the past decades (3). Oral candidiasis represents a serious problem for patients with hematologic malignancies (4). There are many risk factors that make patients susceptible to candidiasis. These risk factors include: a prolonged hospital stay, blood malignancy, treatment with broad-spectrum antibiotics, neoplastic diseases, and repressive treatment of the immune system for organ transplants, intubation and catheter use. Based on the increase in number of patients with such risk factors, incidence of candidiasis also has increased (5). Often these infections happen in patients with hematologic malignancies. This increase depends on the host defense system in the following circumstances: changes in chemotherapy, hematopoietic stem cells transplantation, and abstract radiation use of corticosteroids, cyclosporine and other immunosuppressive agents (6–11). Cancer patients are exposed to localized and diffuse infections, such as oral infections due to illness and chemotherapy-related symptoms, with weakening of the immune system. Cytotoxic chemotherapy, radiation or malignancies themselves are known to compromise the cell-mediated host immunity which plays a significant function in the control of fungal infections. Recent studies express that there is a variation in the incidence of oral yeast colonization and infection amongst different cancer groups (12).

In this study, molecular method, PCR-RFLP, was used to detect candidiasis in the oral mucosa of patients with leukemia and lymphoma in a timely manner in order to prevent their progression to systemic candidiasis. After becoming a systemic candidiasis, it is difficult to diagnose, control the infection in patients with hematological malignancies, especially those with leukemia and lymphoma, and in many cases, this can lead to increase mortality (13).

## MATERIALS AND METHODS

### Patients and specimens collection.

Fifty swabs were collected from the mouths of patients with hematologic malignancies undergoing chemotherapy from the oncology units of the teaching hospitals (Afzalipour, Shahid-Bahonar and Shafa Hospitals) in Kerman, Iran, from March 2017 to February 2018. Patients were from Kerman, Sistan-Baluchestan and Hormozgan provinces of Iran. Fifty samples including 13 patients with acute lymphoid leukemia (ALL); 13 patients, acute myeloid leukemia (AML); 13 patients, chronic lymphoid leukemia (CLL); 5 patients, chronic myeloid leukemia (CML); 5 patients, Hodgkin's lymphoma (HL); 4 patients and non-Hodgkin's lymphoma (NHL); 10 patients.

Samples were collected and transferred to the medical mycology laboratory of Kerman University of Medical Sciences. Mouth swabs were subjected to direct examination with 20% KOH and cultured on Sabouraud's dextrose agar (Merck, Germany) containing chloramphenicol (0.5 μg/mL) (Merck KGa A, Darmstadt, Germany). All isolates were presumptively identified by phenotypic methods such as the color of colonies on CHROMagar *Candida* medium (CHROMagar, India, Cat no: 212961), germ-tube formation in serum and production of chlamydoconidia in corn meal agar (Merck, Germany) with 1% Tween 80.

### Molecular identification: DNA extraction and PCR-RFLP.

The genomic DNA was extracted from yeast cultures using the Exgene cell SV-mini 10^6^-10^1^ DNA tissue kit (GeneAll, South Korea). PCR amplification was performed using universal primers ITS1 (5′-TCCGTAGGTGAACCTGCGG-3′) and ITS4 (5′-TCCTCCGCTTATTGATATGC-3′) in a thermal cycler instrument (Biometra GmbH, Germany) (14). The PCR reaction contained 1 μL of DNA, 10 μL of master mix 2× (YTA 1553, Iran), 1 μL of each primers, and 12 μL of distilled water. The cycling program was: an initial denaturation at 94°C for 5 min followed by 30 cycles of 94°C for 60 s, 60°C for 60 s, 72°C for 120 s and final extension at 72°C for 10 min. The PCR products were electrophoresed in 1% agarose gel in 0.5× TBE buffer and stained with ethidium bromide and then visualized by a Gel Doc system.

### Restriction fragment length polymorphism analysis.

*MspI (HpaII)* (Thermo scientific, USA) restriction enzyme was used for RFLP (Table 1). Digestion with *MSP1* enzyme performed by incubating 10 μL of PCR reaction mixture, 18 μL nuclease-free water, 2 μL 10× buffer Tango and 1 μL of each enzymes in a final reaction volume of 31 μL at 37°C for 3 h. Restriction fragments were separated in 2% agarose gel by electrophoresis in 0.5× TBE buffer, then visualized by a Gel Doc system after staining the with ethidium bromide.

**Table 1.** DNA size for PCR products (bp) of *Candida* spp., after RFLP.

***Candida* spp**	**ITS_1_-ITS_4_**	**MSP_1_**
*C. albicans*	535	238–297
*C. Parapsilosis*	520	520
*C. Tropicalis*	524	184-340
*C. krusei*	510	249-261
*C. guilliermondii*	608	82-155-371
*C. glabrata*	871	314-577
*C. lusitaniae*	383	117-266
*C. kefyr*	721	721

## RESULTS

A total of 50 patients participated in this study, with 14 of them (28%) had positive oral candidiasis.

**Fig. 1.**
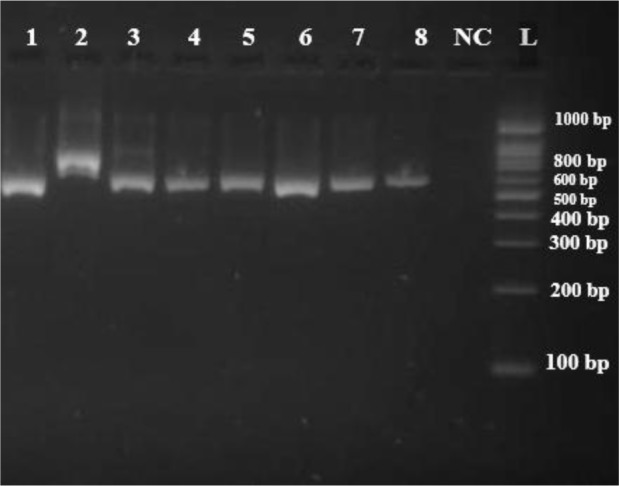
Isolates 1 and 8: Amplification of genomic DNA from clinical isolates using ITS1 and ITS4 primers, NC: Negative Control, L: 100 bp DNA size marker.

**Fig. 2.**
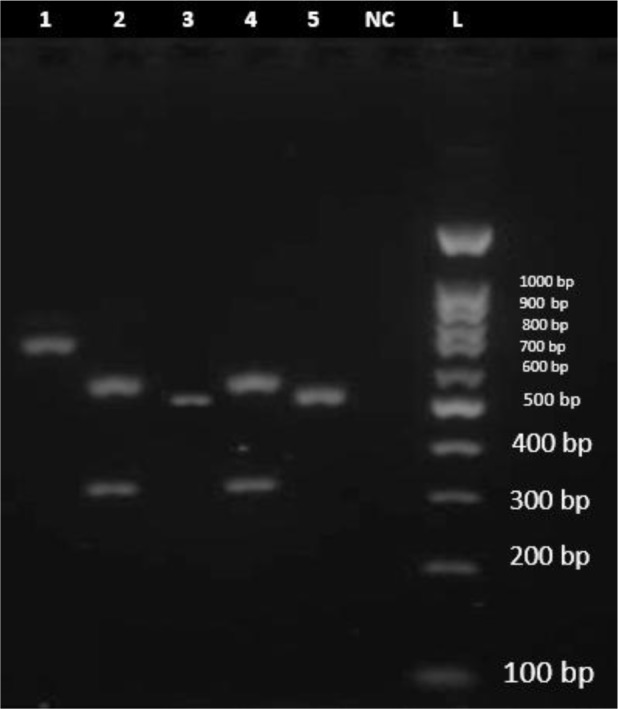
Agarose gel electrophoresis of ITS-PCR products of various pathogenic *Candida* species after digestion with *MspI*. Lanes 1–5: *C. kefyr* (721 bp), *C. glabrata* (314 bp, 577 bp), *C. parapsilosis* (520 bp), *C. glabrata* (314 bp, 577 bp) and *C. parapsilosis* (520 bp) respectively. NC: Negative Control. L: 100 bp DNA size marker.

The species identification was acheived using conventional methods such as growth characteristics on Sabouraud's dextrose agar, color of colony on CHROMagar *Candida* medium, germ tube production and assessing the morphology on corn meal agar. All isolates were re-identified by PCR-RFLP method (Figs. 1, 2).

**Fig. 3.**
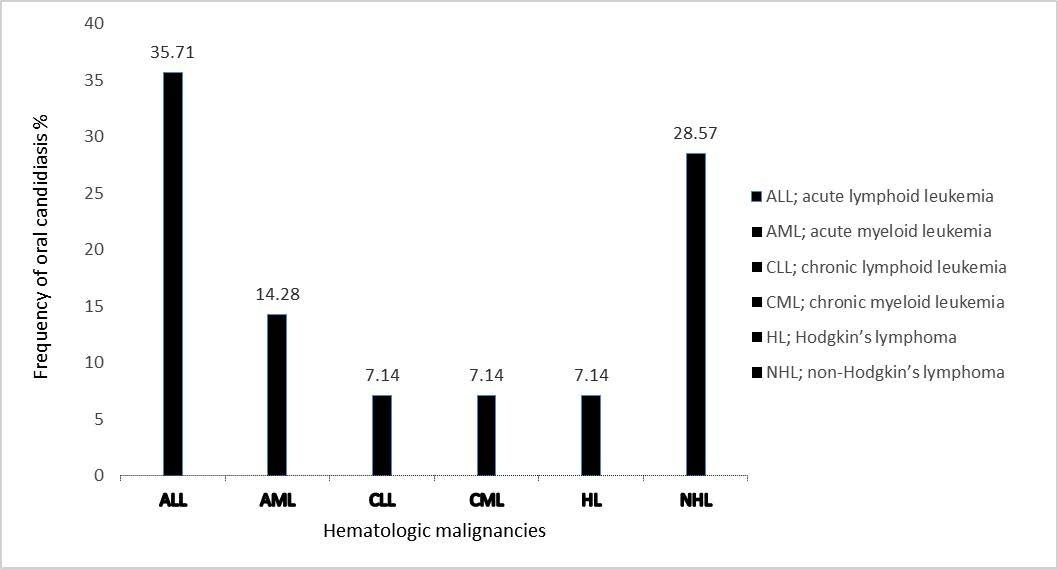
Frequency of oral candidiasis in different types of hematologic malignancies.

*C. albicans* (n=8, 57.14%) was the most common species among them followed by *C. glabrata* (n=2, 14.28%), *C. parapsilosis* (n=2, 14.28%), *C. krusei* (n=1, 7.14%) and *C. kefyr* (n=1, 7.14%). The distribution of *Candida* species isolated from oral candidiasis in patients with hematologic malignancies undergoing chemotherapy, according to clinical presentation and type of cancer, are shown in Fig. 3. The distribution of *Candida* species according to type of hematologic malignancies presented in Table 2. *C. albicans* was the most frequent species isolated in the hematologic malignant disease.

**Table 2.** The frequency distribution of *Candida* species according to type of hematologic malignancies.

***Candida* spp**	**Type of malignancies**						

	**ALL**	**AML**	**CLL**	**CML**	**HL**	**NHL**	**Frequency (%)**
*C. albicans*	3	1	1	1	1	1	8
*C. glabrata*	0	0	0	0	0	2	2
*C. parapsilosis*	1	0	0	0	0	1	2
*C. krusei*	1	0	0	0	0	0	1
*C. kefyr*	0	1	0	0	0	0	1
No *Candida* spp	8	11	4	4	3	6	36
Total	13	13	5	5	4	10	50

ALL, acute lymphoid leukemia; AML, acute myeloid leukemia; CLL, chronic lymphoid leukemia; CML, chronic myeloid leukemia; HL, Hodgkin's lymphoma; NHL, non-Hodgkin's lymphoma

## DISCUSSION

The incidence of opportunistic fungal infections such as oral candidiasis has increased in the past few decades. Several studies have evaluated the incidence of candidiasis, particularly in patients with hematologic malignancies. The most abundant oral fungal infection is candidiasis. In the oral tissues, candidiasis may become visible as pseudomembranous white plaques, erythematous areas, chronic atrophic white plaques, or angular cheilitis (15, 16). Immunosuppression patients are at high risk of life-threatening infection diseases. These patients are more susceptible to systemic infection, particularly those who need chemotherapy for hematologic malignancies (17). Immunocompetent patients scarcely present with oral candidiasis (18). In recent decades, candidiasis has been recognized as a infections affecting in immunosuppression patients with hematologic malignancies (19, 20).

Our study showed the prevalence of oral candidiasis among patients with leukemias and lymphomas was estimated to be 28%. Similar results were reported in the present study by Schelenz et al. (20.5%), Aghadavoudi et al. (29.2%), Lone et al. (22%), and Maheronnaghsh et al. (19.5%) (12, 20–22). Gonzalez et al. on the other hand reported a higher rate of oral candidiasis i.e. 69.35% in cancer patients (4). This result was contradictory with to the findings of our study. This difference in results may be due to possible causes such as climatic conditions, type of nutrition, age and type of treatment received (21, 22). In the present study, the highest prevalence was observed in patients with acute lymphoid leukemia (ALL) and non-Hodgkin's lymphoma (NHL) respectively. The reason for this increase may be the prevalence of these two diseases among children with a weakened immunity system than adults. Various studies have showed that *C. albicans* is the most prevalent candida species isolated from cancer patients (23–25). Oral candidiasis is a frequent complication in pediatric oncology with *C. albicans* is the important etiologic agent, however, there is an association with of other *Candida* species (4). Our data confirm that *C. albicans* was responsible for the majority of the oral candidiasis. Similar to our finding, Badiei et al. and Aghadavoudi et al. showed that the most common candida species in patients with hematologic malignancies is *C. albicans* (23, 24). Also our results are in agreement with Schelenz et al. (12), Lone et al. (26), Maheronnaghsh et al. (27) and Gonzalez et al. (4). Although *C. albicans* continues to be the most common species involved, Shokohi et al. reported other less common *Candida* species, such as *C. orthopsilosis, C. infanticola* and *C. spencermartinsiae*, may be present in clinical specimens obtained from cancer patients (28). Based on our research, there is a higher chance of having a systemic infection in patients with hematological malignancies that have *Candida* colonization (29). Systemic infection can spread to the different organs and increases the mortality.

Oral candidiasis represents a problem for immunosuppressed patients (30). The treatment of hematological malignancies relies heavily on cytotoxic chemotherapy that most often places patients at risk for fungal infections due to disruptions in mucosal barrier integrity and neutropenia (2). Colonizing *Candida* species probably present as reservoir for future systemic candidiasis. In the present study, 28% of immunocompromised patients were colonized by *Candida* species. *C. albicans* was the most prevalent species in patients with leukemia and lymphoma. The information about distribution of candida species can be useful for the management of systemic candidiasis in immunocompromised patients, early diagnosis and empirical antifungal therapies (31).
